# Inferring Population HIV Viral Load From a Single HIV Clinic’s Electronic Health Record: Simulation Study With a Real-World Example

**DOI:** 10.2196/58058

**Published:** 2024-07-03

**Authors:** Neal D Goldstein, Justin Jones, Deborah Kahal, Igor Burstyn

**Affiliations:** 1 Department of Epidemiology and Biostatistics Dornsife School of Public Health Drexel University Philadelphia, PA United States; 2 AIDS Care Group Sharon Hill, PA United States

**Keywords:** HIV, human immunodeficiency virus, viral load, population viral load, electronic health record, EHR, electronic health records, EHRs, electric medical record, EMR, electric medical records, EMRs, patient record, patient record, health record, health records, personal health record, PHR, selection weights, sampling, sampling bias, Bayes

## Abstract

**Background:**

Population viral load (VL), the most comprehensive measure of the HIV transmission potential, cannot be directly measured due to lack of complete sampling of all people with HIV.

**Objective:**

A given HIV clinic’s electronic health record (EHR), a biased sample of this population, may be used to attempt to impute this measure.

**Methods:**

We simulated a population of 10,000 individuals with VL calibrated to surveillance data with a geometric mean of 4449 copies/mL. We sampled 3 hypothetical EHRs from (A) the source population, (B) those diagnosed, and (C) those retained in care. Our analysis imputed population VL from each EHR using sampling weights followed by Bayesian adjustment. These methods were then tested using EHR data from an HIV clinic in Delaware.

**Results:**

Following weighting, the estimates moved in the direction of the population value with correspondingly wider 95% intervals as follows: clinic A: 4364 (95% interval 1963-11,132) copies/mL; clinic B: 4420 (95% interval 1913-10,199) copies/mL; and clinic C: 242 (95% interval 113-563) copies/mL. Bayesian-adjusted weighting further improved the estimate.

**Conclusions:**

These findings suggest that methodological adjustments are ineffective for estimating population VL from a single clinic’s EHR without the resource-intensive elucidation of an informative prior.

## Introduction

There has been increasing interest in using electronic health record (EHR) data as part of public health surveillance efforts [1]. In an interview conducted among local health departments, Comer et al [2] reported 23 such uses, including incidence or prevalence of infectious and chronic diseases, such as diabetes, hepatitis B and C, asthma, and depression, and uptake of disease prevention programs, including vaccination and HIV testing. Uptake of HIV testing is especially relevant and timely given the 2019 US Department of Health and Human Services’ “Ending the HIV Epidemic: A Plan for America” initiative [3]. The plan calls for a 75% reduction in the number of new HIV diagnoses within 5 years and a 90% reduction within 10 years.

To realize this ambitious goal, health departments monitor data on HIV in their jurisdictions. There are a variety of metrics for doing so, including incidence, prevalence, late diagnoses, and viral load (VL), a marker for the success of HIV testing programs and connection to care and treatment. Undetectable VL is the desired outcome in the HIV care continuum because an undetectable VL equates to zero transmission risk, the foundation of treatment as prevention [4]. A hierarchy of aggregated VL measures exist and relate to the natural sampling process that occurs from the source population when individuals are diagnosed (community VL), are connected to care (in-care VL), and have VL measures obtained (monitored VL) [5]. The broadest categorization, population VL, is the most comprehensive measure of the HIV transmission potential. However, population VL cannot be directly measured due to lack of complete sampling of the population of people living with HIV as well as lack of complete or recent VL data among those diagnosed [5]. Despite its utility and appeal, the measure has notable challenges, including population selection, varying definitions and calculations, and complete and accurate surveillance [6]. These issues may have led to the decline in its use following its introduction in 2009. Nevertheless, population VL—if quantifiable—is a useful latent measure of transmission potential and quality of HIV care and treatment in a specific geographic area. Even a biased measure can be useful if it can be calibrated to a less biased or an unbiased measure. For example, one contemporary paper using data from the 2010s investigated community VL and HIV incidence in South Carolina and found that community VL disparities mirrored disparities in HIV access to care for nonprioritized groups including women, rural populations, and heterosexual transmission [7].

Absent complete (or a representative random) sampling of a population of people living with HIV, one may turn to EHRs from various clinics to estimate population VL. A given health department might wish to know the distribution of VL among people living with HIV in its jurisdiction but only have a single HIV care program that serves the community. As such, the ability to estimate population VL from a single EHR may be of value. In fact, researchers have previously demonstrated how EHR data can improve the accuracy of HIV surveillance programs [8]. However, use of EHRs for these purposes faces methodological challenges, including ambiguous catchment [9]. A given EHR can be expected to over- or under-sample with respect to characteristics of people living with HIV (eg, health, income, race, age, distance to clinic). We sought to investigate the feasibility of imputing population VL from a single EHR and under what conditions this may be possible.

## Methods

### Creation of the Synthetic Data Set and Clinics

To establish the feasibility of recovering the true population VL from a single clinic’s EHR, we would need both clinic-level VL EHR data as well as the VL from the source population, data which are difficult to obtain as this would require measuring VL among those unaware of their HIV status as well as those not engaged in care. In lieu of this, we created a hypothetical synthetic source population: This population can be considered a large urban area in the United States with a population size of 1,000,000 people and 1% HIV seropositivity, or 10,000 people living with HIV. We defined 3 demographic strata for the population, as follows: age: <35 years, 35-44 years, 45-54 years, >54 years; gender: male, female; race/ethnicity: non-Hispanic White, non-Hispanic Black or African American, Hispanic or Latino. These categories were not meant to be inclusive of all risk groups but rather commonly reported groups for calibrating VL.

The demography of people living with HIV was randomly sampled from a uniform distribution with probabilities informed from the Centers for Disease Control and Prevention (CDC) 2020 HIV Surveillance Report [10]. Specifically, approximately 75% of the population was set to male, and 25% was set to female. Age distributions were as follows: 18% <35 years, 19% 35-44 years, 24% 45-54 years, and 39% >54 years. Race/ethnicity distributions were as follows: 33% White, 45% Black or African American, and 23% Hispanic or Latino. VL was randomly sampled from a log-normal distribution with a log_10_ geometric mean of 3.65 (4449 copies/mL) and a log_10_ SD of 1.2. The mean was informed from the measured community VL from the San Francisco, CA HIV/AIDS Case Surveillance System for 2005-2008 [11], and the SD was informed from the CDC’s guidance document on community VL [5]. VL was adjusted jointly across the demographic strata by multiplying the VL by a randomly sampled probability obtained from a normal distribution with the following means and accompanying SD of 10%: –1% male and +18% female; +21% <35 years, –10% 35-44 years, –26% 45-54 years, and –26% >54 years; –10% White, +13% Black or African American, and +15% Hispanic or Latino. These adjustments were informed from differences observed in VL in the San Francisco surveillance data [11].

To simulate the HIV care continuum from this source population, we set approximately 10% of the population as unaware of their HIV status. This group was more likely to be younger, male, and Black or African American based on a study of concurrent HIV and AIDS diagnosis in San Francisco [12]. Among those aware of their status, we created an “in care” group in which approximately 72% of those in care would be virally suppressed (<200 copies/mL), mirroring the 2021 San Francisco HIV epidemiology annual report [13], although we stress that our primary intention is not to replicate San Francisco surveillance data but rather create a hypothetical urban population. Sampling the “in care” group in this manner resulted in an average 20% of the aware group also being in care.

Finally, to isolate the effects of various sampling mechanisms, we created 3 HIV clinics with differing catchments. Clinic A was sampled directly from the source population, Clinic B was sampled from those aware of their HIV status, and Clinic C was sampled from those in care. Each clinic contained 250 active patients oversampled by male sex, White race, and age ≥45 years. The demographic composition of each clinic was set to reflect observed patterns of retention in HIV care [14] and to yield an EHR in which the mean VL differed from the source population. We created 1000 versions of each clinic to account for random variability.

### Creation of the Catchment Sampling Weights and Weighted Analysis

Let *K* be the size of the source population, *V* be the number of people aware of their HIV status, *N* be the number of people in care, and *S* be the number of patients in care at a clinic. We can estimate the catchment sampling weight using equation 1:

*W* = 1 / *Beta*((*S* + 1), (*N* + 1 - *S*)) **(1)**

In this equation, N arises from *Binomial*(*N*/*K*, *K*), where *N*/*K* is the prevalence of people living with HIV and in care in the source population. Weights are calculated per the demographic strata enumerated earlier that related to a clinic’s catchment (ie, race, age, and gender) such that *V*, *N*, *S*, and *W* are all calculated separately for each stratum. The final sample weight is obtained for each person by multiplying the corresponding stratum-specific weights.

To allow for the possibility of weight misspecification when they are not estimated appropriately, for example due to an ambiguous catchment, we transformed *W* as outlined in equation 2:

*P_biased_* = log(*P*/(1-*P*)) + b*log(VL) **(2)**

In this equation, *P* is the inverse of *W*, that is, the individual selection probability of being in the clinic, and consequently, the inverse of *P_biased_* is the misspecified (biased) weights. The coefficient “b” is the bias factor and was set to 0.1, a conservative starting point that would still meaningfully shift the weights. Under equation 2, a positive bias factor demonstrates the scenario whereby individuals with higher VLs are less likely to be sampled in the clinic, but, unbeknownst to the researchers, the catchment model does not identify them as such. Consequently, this bias factor down-weighted their contribution in the weighted analysis by a factor of 0.1, when they should have been up-weighted. Larger bias factors would create greater weight misspecification, albeit with the same conclusions.

We simulated 1000 of the unbiased and biased weights per participant, then calculated the population geometric mean (GM) VL for each clinic, where GM=exp(mean(log_10_(VL))). We also calculated the unweighted GM and took the root mean squared error (RMSE) between the weighted and unweighted measures. The final calculations are thus based on the 1000 weights for each of the 1000 clinic As, 1000 clinic Bs, and 1000 clinic Cs. Our target estimand was the median and 95% interval of each clinic’s GM distribution.

### Postweighting Bayesian Adjustment

Following the weighted analysis, we conducted a Bayesian analysis with the expectation that this would further improve our ability to impute the population VL from a given clinic. This approach is analogous to that taken by others who treated weighted observations as “data” that enter the likelihood part of the Bayesian computation [15]. For this analysis, we assumed the true mean and variance were unknown and specified a Normal-Gamma conjugate prior, although, since our focus was only on the posterior mean, the calculations became simplified. The prior mean (µ_0_) was informed by the San Francisco HIV/AIDS Case Surveillance System, namely log_10_ GM VL of 3.65. As a starting point for the prior sample size (n_0_), we took the perspective of a clinic’s population’s VL measured at a previous time point (ie, available before the observed VL data used in the weighted analysis). For example, one might posit that such data were collected immediately upon diagnosis as opposed to routine monitoring during antiretroviral therapy. Following our weighted analysis, these observed measurements have a logarithmic mean of 
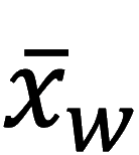
 and effective sample size, n_w_=ΣW for each of the 1000 clinic samples. The posterior logarithmic mean of the population VL (μ_n_) conditional on posterior variance is specified in equation 3.

μ_n_ = ((n_0_×μ_0_) + n_w_×
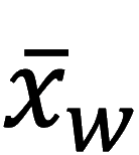
) / (n_0_ + n_w_) **(3)**

To examine the influence of the prior sample size, we operationalized n_0_ in 3 ways: 0.25×n_w_, 0.5×n_w_, and 2×n_w_. Additionally, to reflect the earlier scenario of the prior data collected upon diagnosis, we conducted a final analysis for clinic C where µ_0_ was informed from clinic B’s weighted mean and the more conservative n_0_=0.25*n_w_. As before, these calculations were performed for each of the 1000 clinic weights for each of the 1000 clinics.

### Real-World Clinic Data Set

As an applied demonstration of our methods, we obtained the most recently available VL on active patients retained in care for HIV at the Holloway Community Program at ChristianaCare (Wilmington, DE). Patients’ age, race, and gender were coded using the same categories defined earlier for our synthetic population. Denominators needed for the catchment model were obtained from the US Census Bureau 2021 American Community Survey [16] population sizes for Delaware (the presumed catchment of the Holloway program) and the Delaware Integrated HIV Prevention and Care Plan for 2022-2026 that includes statewide surveillance data as of 2019 [17]. Using the procedures outlined earlier, we estimated the population VL from the clinic data; however, as we did not have access to historic unbiased estimates of VL for this jurisdiction, we used the prior as described in our synthetic analysis. To further acknowledge uncertainty in the prior mean (µ_0_), we conducted a sensitivity analysis with µ_0_ modified in 3 ways (0.25×µ_0_, 0.5×µ_0_, and 2×µ_0_) and repeated the Bayesian adjustment across the 3 prior sample sizes.

All analyses were performed in R version 3.6.3 (R Foundation for Statistical Computing). Analytic codes are available for download from [18]. HIV VL point estimates and 95% intervals are presented on a linear scale in the main text and a logarithmic scale in Multimedia Appendices 1-5.

## Results

### Synthetic Population and Clinics

Each clinic was approximately 93% male; 4% <35 years, 5% 35-44 years, 40% 45-54 years, and 51% >54 years; and 81% White, 13% Black or African American, and 6% Hispanic or Latino (Table 1).

**Table 1:** Characteristics of the synthetic population and clinics as well as the real-world cohort from the Holloway Community Program at ChristianaCare (Wilmington, DE).

Characteristic		Synthetic EHRs^a,b^					Real-world EHR
		Population^c^ (n=10,000)	Clinic A^d^ (n=250)	Clinic B^e^ (n=250)	Clinic C^f^ (n=250)	Holloway (n=1807)	
**Age (years), n (%)**							
	<35	1817 (18.2)	12 (4.8)	10 (4)	10 (4)	278 (15.4)	
	35-44	1819 (18.2)	12 (4.8)	12 (4.8)	15 (6)	299 (16.5)	
	45-54	2727 (27.3)	97 (38.8)	98 (38.2)	96 (38.4)	332 (18.4)	
	>54	3634 (36.3)	129 (51.6)	130 (52)	129 (51.6)	898 (49.7)	
**Gender, n (%)**							
	Female	2497 (25)	17 (6.8)	17 (6.8)	19 (7.6)	558 (30.9)	
	Male	7503 (75)	233 (93.2)	233 (93.2)	213 (92.4)	1249 (69.1)	
**Race/ethnicity, n (%)**							
	Non-Hispanic Black or African American	4446 (44.5)	30 (12)	27 (10.8)	32.5 (13)	1128 (62.4)	
	Non-Hispanic White	3332 (33.3)	205 (82)	207 (82.8)	200 (80)	514 (28.4)	
	Hispanic or Latino	2220 (22.2)	15 (6)	15 (6)	18 (7.2)	165 (9.1)	
Viral load (copies/mL), geometric mean		3996	3147	3108	173	41	
Viral load, log_10_ geometric mean		3.60	3.50	3.49	2.24	1.61	

^a^EHR: electronic health record.

^b^The 3 synthetic clinic electronic health records (n=250 per clinic) were sampled from a source population of people living with HIV (n=10,000) and were oversampled by male sex, White race, and age ≥45 years.

^c^Synthetic population results given as the median values from 1000 hypothetical clinics.

^d^Sampled directly from the source population.

^e^Sampled from a subset of the source population based on diagnosed HIV.

^f^Sampled from a subset of the source population based on retention in care.

**Figure 1:**
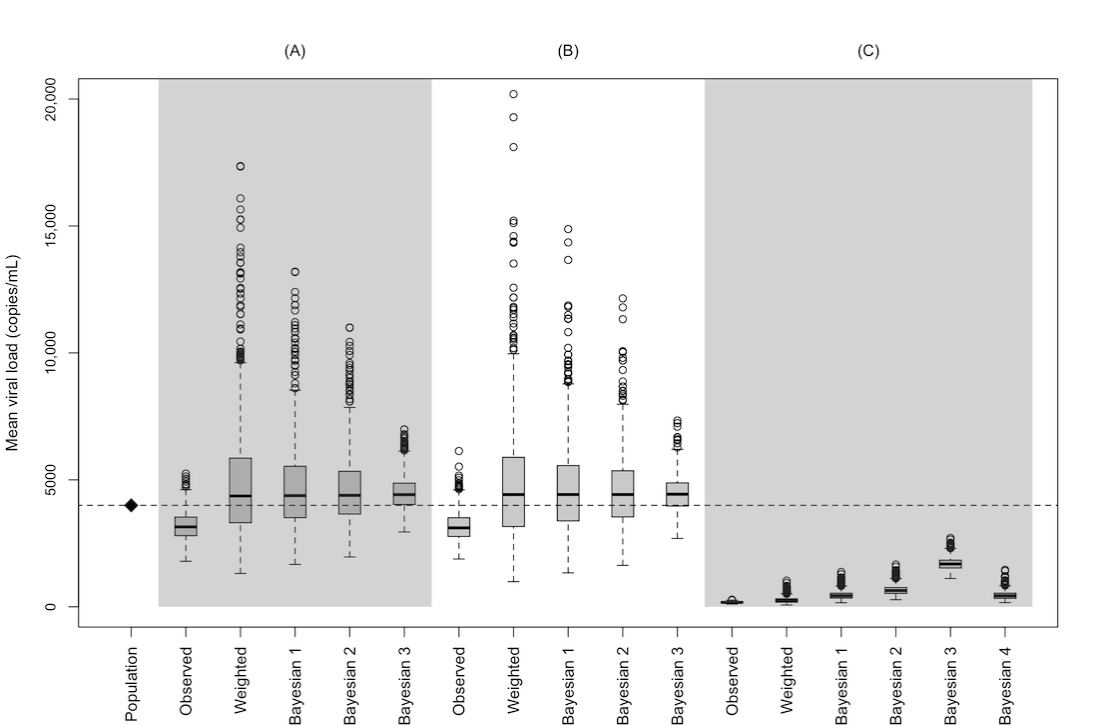
Comparison of the distribution of the geometric mean HIV viral load for 3 clinic electronic health records (n=250 per clinic) sampled from a synthetic source population of people living with HIV (n=10,000), with results representing 1000 hypothetical clinicals each with 1000 sampling weight adjustments based on sampling from the source population (A) directly, (B) based on diagnosed HIV, or (C) based on retention in care (all 3 oversampled by male sex, White race, age ≥45 years). Bayesian 1: prior sample size of 25% of the weighted clinic sample size; Bayesian 2: prior sample size of 50% of the weighted clinic sample size; Bayesian 3: prior sample size of 200% of the weighted clinic sample size; Bayesian 4: prior mean informed from weighted clinic B estimates.

Figure 1 contrasts the observed, weighted, and Bayesian adjusted VLs comparing the clinics to the population (see Multimedia Appendix 1 for logarithmic results). Across the 1000 simulations, the median GM population VL was 3996 (95% interval 3780-4214) copies/mL. For each clinic A, B, and C, the median GM VL point estimates and 95% intervals were 3147 (95% interval 2294-4301), 3108 (95% interval 2216-4383), and 173 (95% interval 123-240) copies/mL, respectively. Following weighting, the estimates moved in the direction of the population value with correspondingly wider 95% intervals as follows: clinic A: 4364 (95% interval 1963-11,132) copies/mL; clinic B: 4420 (95% interval 1913-10,199) copies/mL; clinic C: 242 (95% interval 113-563) copies/mL. Bayesian adjustment resulted in a shrinking of intervals, depending on the prior sample size, where the large sample size resulted in tighter intervals, and clinic C had a notable shift in point estimates toward the population mean. With a 25% of the clinic prior sample size, the posterior estimates were 433 (95% interval 236-851) copies/mL; with a 50% of the clinic prior sample size, the posterior estimates were 639 (95% interval 384-1120) copies/mL; and with a 200% of the clinic prior sample size, the posterior estimates were 1685 (95% interval 1307-2231) copies/mL. When using the weighted clinic B estimates to inform the prior for clinic C, we also noted an improvement in estimating the population mean, with posterior estimates of 432 (95% interval 230-889) copies/mL.

**Figure 2:**
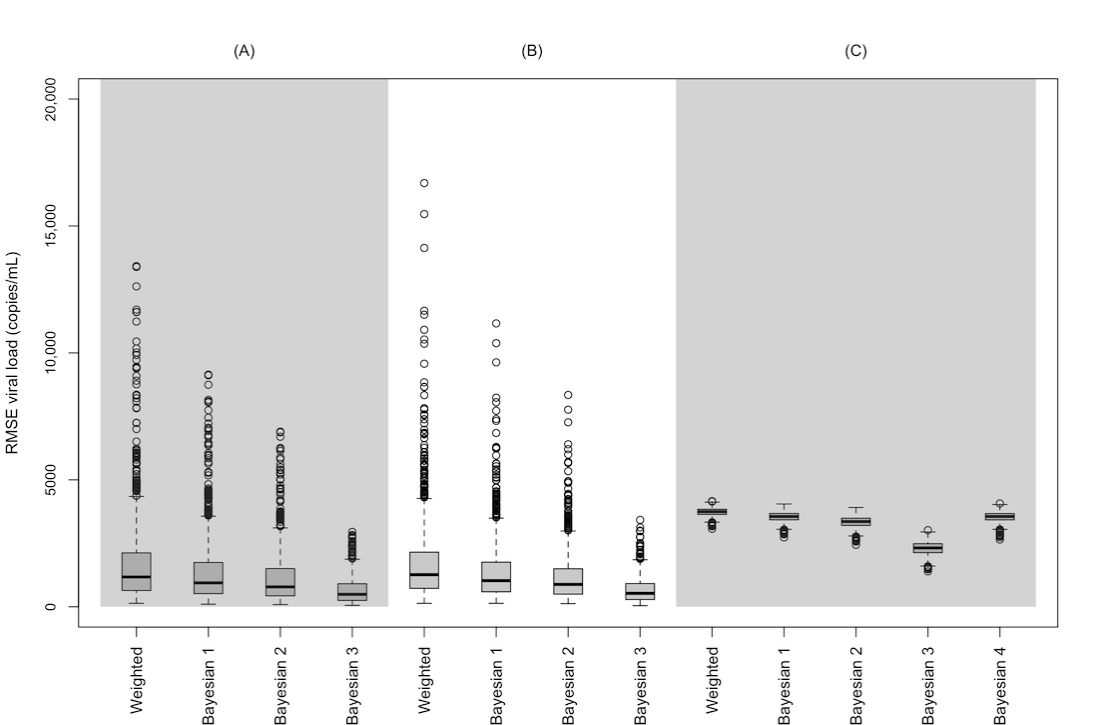
Comparison of the root mean squared error (RMSE) of the geometric mean HIV viral load for 3 clinic electronic health records (n=250 per clinic) sampled from a synthetic source population of people living with HIV (n=10,000), with results representing 1000 hypothetical clinicals each with 1000 sampling weight adjustments based on sampling from the source population (A) directly, (B) based on diagnosed HIV, or (C) based on retention in care (all 3 oversampled by male sex, White race, age ≥45 years). Bayesian 1: prior sample size of 25% of the weighted clinic sample size; Bayesian 2: prior sample size of 50% of the weighted clinic sample size; Bayesian 3: prior sample size of 200% of the weighted clinic sample size; Bayesian 4: prior mean informed from weighted clinic B estimates.

**Figure 3:**
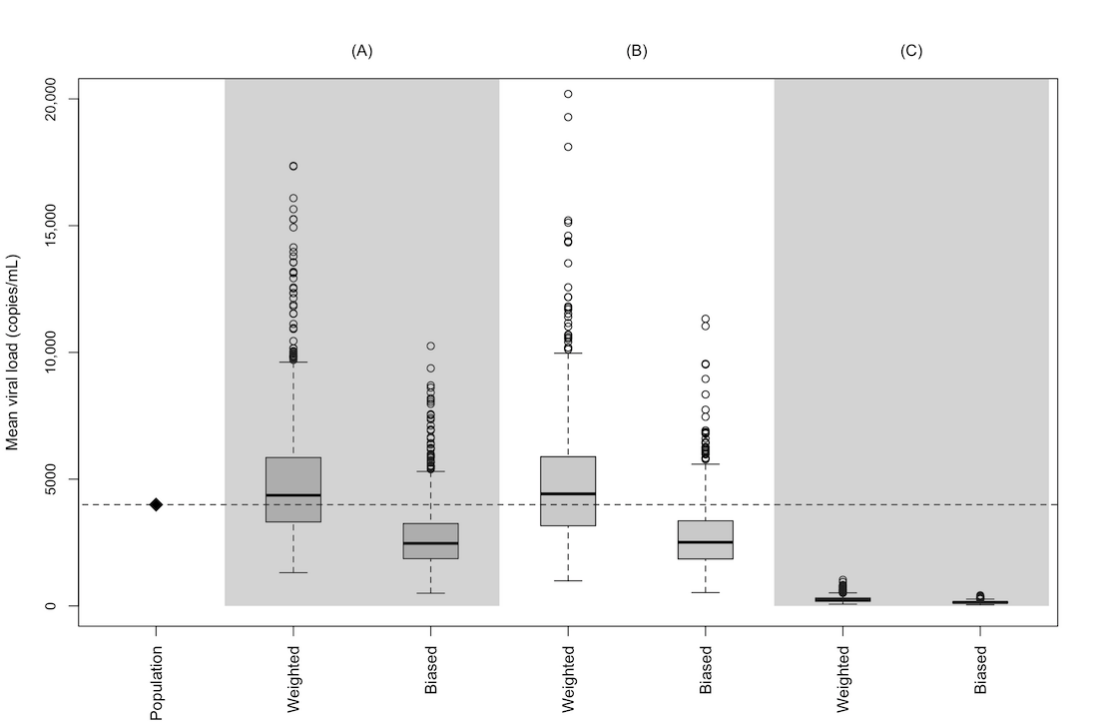
Comparison of weight misspecification in the weighted geometric mean HIV viral load for 3 clinic electronic health records (n=250 per clinic) sampled from a synthetic source population of people living with HIV (n=10,000), with results representing 1000 hypothetical clinicals each with 1000 sampling weight adjustments based on sampling from the source population (A) directly, (B) based on diagnosed HIV, or (C) based on retention in care (all 3 oversampled by male sex, White race, age ≥45 years).

Figure 2 depicts the averaged RMSE for each clinic for each weighting strategy (see Multimedia Appendix 2 for logarithmic results). RMSE was greatest in the purely weighted analyses, with median errors and 95% intervals for each clinic as follows: clinic A: 1174 (95% interval 288-7261) copies/mL; clinic B: 1265 (95% interval 261-6369) copies/mL; and clinic C: 3745 (3385-4018) copies/mL. RMSE was lowest in the Bayesian analysis that followed weighting with the larger prior sample size, as follows: clinic A: 490 (95% interval 92-2026) copies/mL; clinic B: 528 (95% interval 96-1884) copies/mL; and clinic C: 2319 (95% interval 1773-2747) copies/mL. Figure 3 shows the impact of the weight misspecifications (see Multimedia Appendix 3 for logarithmic results). As expected, the biased weight systematically down-weighted higher VL individuals when they should have been up-weighted, as might occur based on an inaccurate catchment model where individuals with higher VLs were less likely to be sampled in the clinic.

### Holloway Community Program Clinic

The 2021 population in Delaware was 1,003,384. For each demographic stratum, the populations had the following characteristics: age (<35 years: 420,844; 35-44 years: 122,088; 45-54 years: 115,300; >54 years: 345,152), gender (male: 485,908; female: 517,476), and race/ethnicity (non-Hispanic White: 595,212; non-Hispanic Black or African American: 205,217; Hispanic or Latino: 101,213; other: 101,742). As of 2019, there were an estimated 3841 people living with HIV; 2984 were in care, and 857 were not in care. For each demographic stratum among those in care, the populations had the following characteristics: age (<35 years: 394; 35-44 years: 432; 45-54 years: 703; >54 years: 1455), gender (male: 2125; female: 859), and race/ethnicity (non-Hispanic White: 958; non-Hispanic Black or African American: 1725; Hispanic or Latino: 222; other: 79).

There were 1807 active patients in the Holloway Community Program with a resulted VL test as of the date of EHR data extraction. The GM VL of the clinic was 41, and the geometric SD was 190,261 copies/mL; 1656 of the 1807 (91.6%) patients were virally suppressed (<200 copies/mL). Additional characteristics may be found in Table 1.

**Figure 4:**
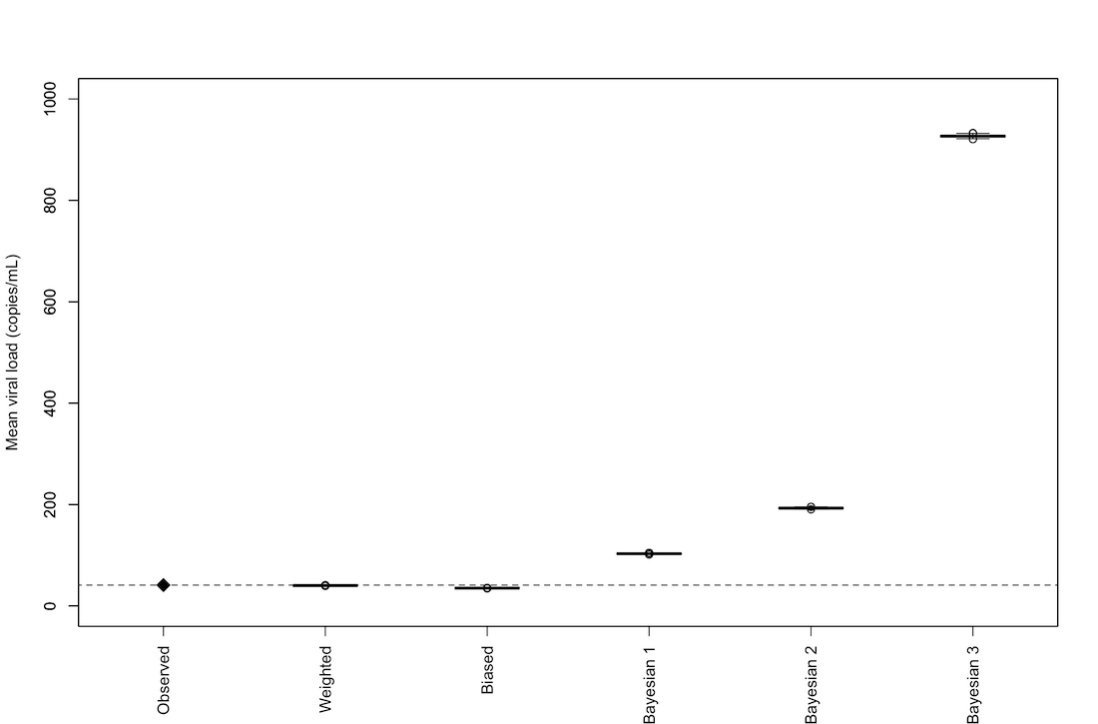
Inferred population geometric mean HIV viral load for Delaware based on active patients retained in care at the Holloway Community Program at ChristianaCare (Wilmington, DE), with results representing 1000 sampling weight adjustments. Bayesian 1: prior sample size of 25% of the weighted clinic sample size; Bayesian 2: prior sample size of 50% of the weighted clinic sample size; Bayesian 3: prior sample size of 200% of the weighted clinic sample size.

Figure 4 presents the inferred population VL measure from the clinic’s EHR (see Multimedia Appendix 4 for logarithmic results). The weighting-only adjustment had negligible impact compared with the unweighted estimate, while the biased weights shifted the estimates slightly lower to a median of 35 copies/mL. The biased weight systematically down-weighted higher VL individuals when they should have been up-weighted, as might occur based on an inaccurate catchment model where individuals with higher VLs were less likely to be sampled in the clinic. Meanwhile, the Bayesian adjustment moved the weighted estimate from 40 copies/mL to a median of 103 copies/mL with the 25% prior sample size, to 193 copies/mL with the 50% prior sample size, and to 926 copies/mL with the 200% prior sample size. Results were sensitive to the assumption about the informative prior’s mean (Multimedia Appendix 5).

## Discussion

Using a synthetic population, we observed that recovery of population VL from a single center’s monitored VL was hampered when a historic measurement or informed guess at the prior population VL was unavailable. In other words, good VL data are preferred to methodological adjustments of incomplete data.

Community VL, calculated from individuals who have been diagnosed with HIV, has been used to generalize risk of HIV transmission and evaluate retention in care and viral suppression [6]. However, as mentioned in the Introduction, this measure has several shortcomings. First, it will almost always result in underestimated VL, as individuals who are unaware of their HIV status will likely have higher VLs. Relatedly, timing of the individual VL measure may also impact the community estimate, as VL will fluctuate over time (eg, acute vs chronic infection). Second, there may be issues with defining the specific geographic area of the community and whether this population is “closed.” Although closed communities would allow for a more accurate community VL measurement, the applicability and feasibility are hindered in the real world by population migration. Third, sampling bias may be present when there is a high prevalence of undiagnosed people living with HIV [6]. In these situations, the use of the population VL may be more appropriate for reflecting transmission potential should we be able to impute data for those undiagnosed or not retained in care. To address these limitations, alternate metrics have been proposed, such as the prevalence of viremia based on viral suppression [19]. As such, researchers have adopted alternative community-level VL measures that reflect the prevalence of HIV in the community as well as distinguishing between those who are virally suppressed and those who have a high VL and are more likely to contribute to community spread [6,19]. The methods we have demonstrated can readily be adapted to other HIV measures where a weighted mean may be desired, such as CD4 cell counts among people living with HIV for a given jurisdiction. Regardless of the metric used, there is still risk of ecologic fallacy at the aggregate level wherein a higher population VL may not correspond to higher individual transmission risk when prophylaxis is common.

Others have acknowledged the important challenge of the use of EHR data for population inference when health care–seeking behavior and access to care impact representativeness. EHR-based studies are susceptible to issues of confounding, information bias, and selection bias [9]. Bower et al [20] demonstrated how selection into an EHR is not random and recommended techniques such as sampling (poststratification) weights and propensity scoring and inverse probability weighting (IPW) to adjust estimates, in their case, of cardiovascular disease risk. Flood et al [21] used EHR data to estimate childhood obesity and found that the application of sampling weights to their data allowed estimates to be comparable to a nationally representative survey. Goldstein et al [22] used IPW to adjust for presumed selection bias in a single-center EHR-based study when exposure and outcome relate to catchment. It is worth delineating how these 2 complementary strategies—sampling/poststratification weighting versus propensity scoring/IPW—differ in EHR research.

The IPW approach requires specification of a probability model (ie, the propensity score) for selection into the EHR from the source population, conditioned on measured characteristics related to this process. However, this demands the EHR capture relevant details on the catchment process, or those data can be readily linked, and EHRs are well-known to lack data on epidemiological determinants [23]. On the other hand, using a sampling weight assumes we have access to the denominators from which the EHR data are sampled. One such source of data we have used are census estimates, which can be stratified by factors relating to catchment and tuned to the local environment. The challenge with this approach is that, in practice, we may not know all the characteristics defining catchment process, the census might not capture those characteristics, or there may be ambiguous geography. Indeed, catchment is a multifactorial and sometimes nebulous process related to health care availability, accessibility, affordability, accommodation, and acceptability [24]. One potential way to gain insight into catchment is to compare EHR data with census data to see which characteristics are over- or under-represented for a given geographic area defined by the clinic. If the census lacks data on catchment-relevant factors but the EHR captures these details (eg, sexual orientation), this may favor the IPW approach.

Another important limitation of our approach was our construction of the sampling weights. We assumed a simple random sample within each catchment stratum to calculate the sampling weights. In our synthetic population, this was known with certainty, although we blinded ourselves to this oracle view by not retaining the selection probabilities during the data generation process but rather relying on our catchment model. However, as exemplified in our biased weighted analysis and the real-world clinic data set, the catchment stratum may be uncertain and, in our case, presumably underestimated population VL. Many extensions exist to improve weighting approaches, such as raking, which we did not evaluate herein [25]. We also observed a decrease in precision—widening of intervals—when comparing the weighted versus unweighted results. This has been termed the bias-variance tradeoff, where improved accuracy may be accompanied by worsened precision [26].

A particular strength to our approach is the straightforward implementation and Bayesian adjustment that can be carried out with minimal programming ability. The included source code [18] can serve as a starting point. More complicated cluster survey designs may also benefit from Bayesian methods [27,28]. Bayesian analysis requires careful deliberation over which priors may be most appropriate. Informative priors are useful and straightforward, but obtaining unbiased estimates of VL can be prohibitively expensive for some jurisdictions, and measures obtained in one jurisdiction may not be exchangeable with another. Indeed, we observed that our real-world application was sensitive to the choice of prior. Nonetheless, even a small unbiased survey can dramatically reduce RMSE and thus may be justified. This would have to be done only once to seed Bayesian prospective surveillance of population VL. These methods can be adapted to other aggregated measures of disease prevalence, for both research and practice purposes, especially if an historic prior estimate is available.

Health departments have expressed interest in using EHR data for many community health measures that can help inform resource allocation and public health decision-making in different contexts. Comer et al [2] identified 23 of these; hepatitis B and C infection was a high priority measure and one in which previous surveys such as the National Health and Nutrition Examination Survey [29,30] can serve as an informative prior. If, for example, a focal outbreak of hepatitis C is detected from an EHR, this could suggest targeted treatment and prevention efforts to cure infection and reduce future transmission.

In short, we observed that methodological adjustments were ineffective to recover the true population VL in our data without prior knowledge of what this value may be. Further validation using real-world EHR data from diverse clinical settings is needed to confirm this finding. Should such prior data be available, then it may be possible to infer population characteristics from a biased clinic sample in the EHR. Moving forward, we encourage those with access to population-based surveys of community health metrics—especially at subnational levels—to continue to disseminate these data to enable epidemiologic methods such as ours.

## Appendix

Multimedia Appendix 1 Comparison of the distribution of the logarithmic geometric mean HIV viral load (VL) for three clinic electronic health records (n=250 per clinic) sampled from a synthetic source population of people living with HIV (n=10,000). Clinic A was sampled directly from the source population, whereas clinics B and C were sampled from a subset of the source population based on diagnosed HIV (clinic B) or retention in care (clinic C). All synthetic clinics oversampled by male sex, White race, and 45 years of age or older. Results represent 1,000 hypothetical clinics each with 1,000 sampling weight adjustments. 1 Prior sample size of 25% of the weighted clinic sample size. 2 Prior sample size of 50% of the weighted clinic sample size. 3 Prior sample size of 200% of the weighted clinic sample size. 4 Prior mean informed from weighted clinic B estimates.

Multimedia Appendix 2 Comparison of the root mean squared error (RMSE) of the logarithmic geometric mean HIV viral load (VL) for three clinic electronic health records (n=250 per clinic) sampled from a synthetic source population of people living with HIV (n=10,000). Clinic A was sampled directly from the source population, whereas clinics B and C were sampled from a subset of the source population based on diagnosed HIV (clinic B) or retention in care (clinic C). All synthetic clinics oversampled by male sex, White race, and 45 years of age or older. Results represent 1,000 hypothetical clinics each with 1,000 sampling weight adjustments. 1 Prior sample size of 25% of the weighted clinic sample size. 2 Prior sample size of 50% of the weighted clinic sample size. 3 Prior sample size of 200% of the weighted clinic sample size. 4 Prior mean informed from weighted clinic B estimates.

Multimedia Appendix 3 Comparison of weight misspecification in the weighted logarithmic geometric mean HIV viral load (VL) for three clinic electronic health records (n=250 per clinic) sampled from a synthetic source population of people living with HIV (n=10,000). Clinic A was sampled directly from the source population, whereas clinics B and C were sampled from a subset of the source population based on diagnosed HIV (clinic B) or retention in care (clinic C). All synthetic clinics oversampled by male sex, White race, and 45 years of age or older. Results represent 1,000 hypothetical clinics each with 1,000 sampling weight adjustments. The biased weight systematically down-weighted higher VL individuals when they should have been up-weighted, as might occur based on an inaccurate catchment model where individuals with higher VLs were less likely to be sampled in the clinic.

Multimedia Appendix 4 Inferred population geometric mean HIV viral load (VL) for Delaware based on active patients retained in care at the Holloway Community Program at ChristianaCare (Wilmington, DE). Results represent 1,000 sampling weight adjustments. The biased weight systematically down-weighted higher VL individuals when they should have been up-weighted, as might occur based on an inaccurate catchment model where individuals with higher VLs were less likely to be sampled in the clinic. 1 Prior sample size of 25% of the weighted clinic sample size. 2 Prior sample size of 50% of the weighted clinic sample size. 3 Prior sample size of 200% of the weighted clinic sample size.

Multimedia Appendix 5 Sensitivity analysis of inferred population geometric mean HIV viral load (VL) for Delaware based on active patients retained in care at the Holloway Community Program at ChristianaCare (Wilmington, DE). Results represent 1,000 Bayesian sampling weight adjustments. Sensitivity analysis compared three alternate specifications of the prior mean for VL: 25%, 50%, and 200% of the original specification (m0). Prior sample size was varied three ways: 25%, 50%, and 200% of the weighted clinic sample size.

## Abbreviations

CDC: Centers for Disease Control and Prevention
EHR: electronic health record
GM: geometric mean
IPW: inverse probability weighting
PLWH: people living with HIV
RMSE: root mean squared error
VL: viral load
