# Supplementary figures and images for: Inferring Population HIV Viral Load From a Single HIV Clinic’s Electronic Health Record: Simulation Study With a Real-World Example
Source: Online J Public Health Inform. 2024 Jul 3;16:e58058. doi: 10.2196/58058 (PMC11255534; doi:10.2196/58058)

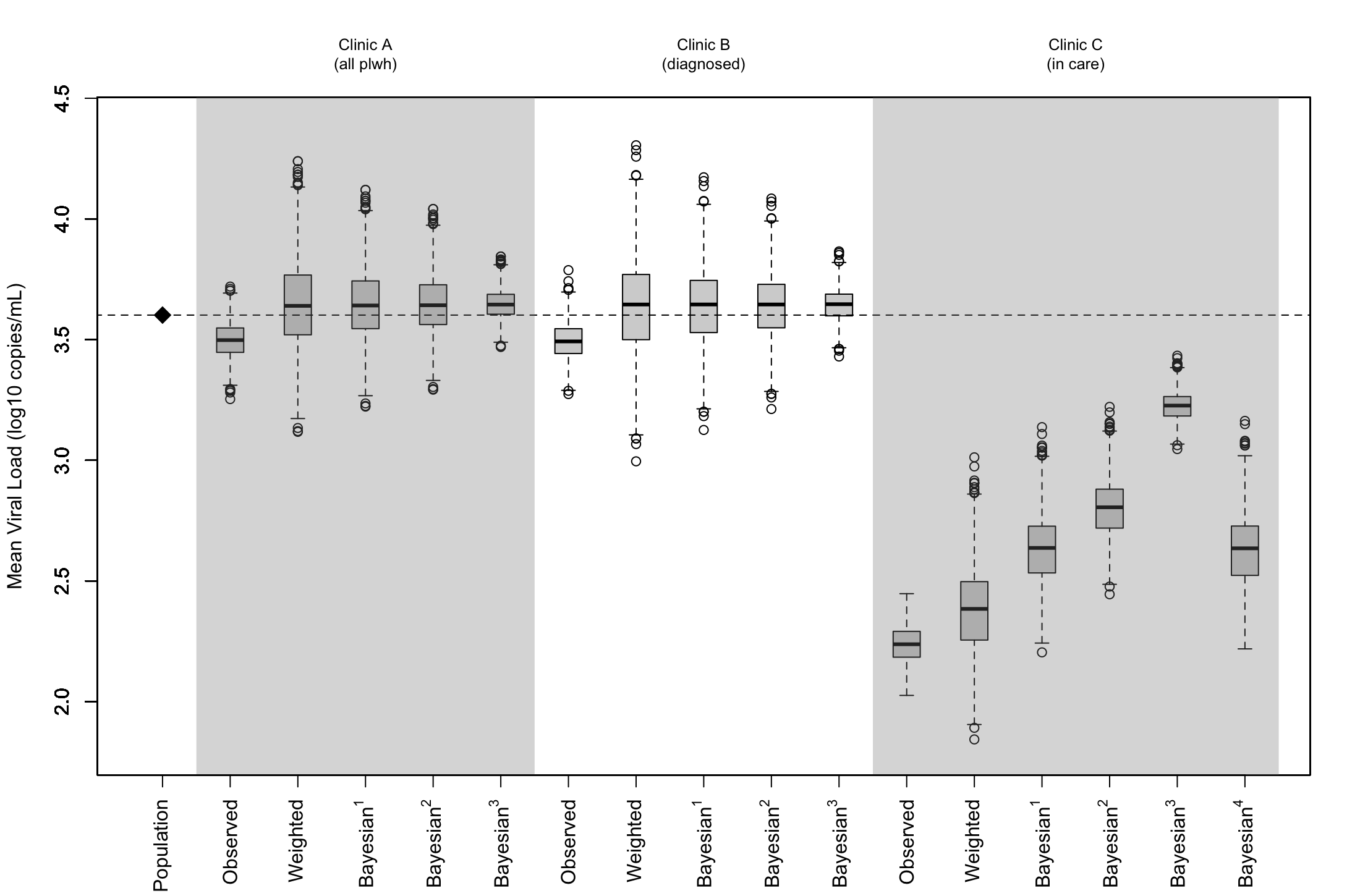

Supplement: Multimedia Appendix 1 [file ojphi_v16i1e58058_app1.png]

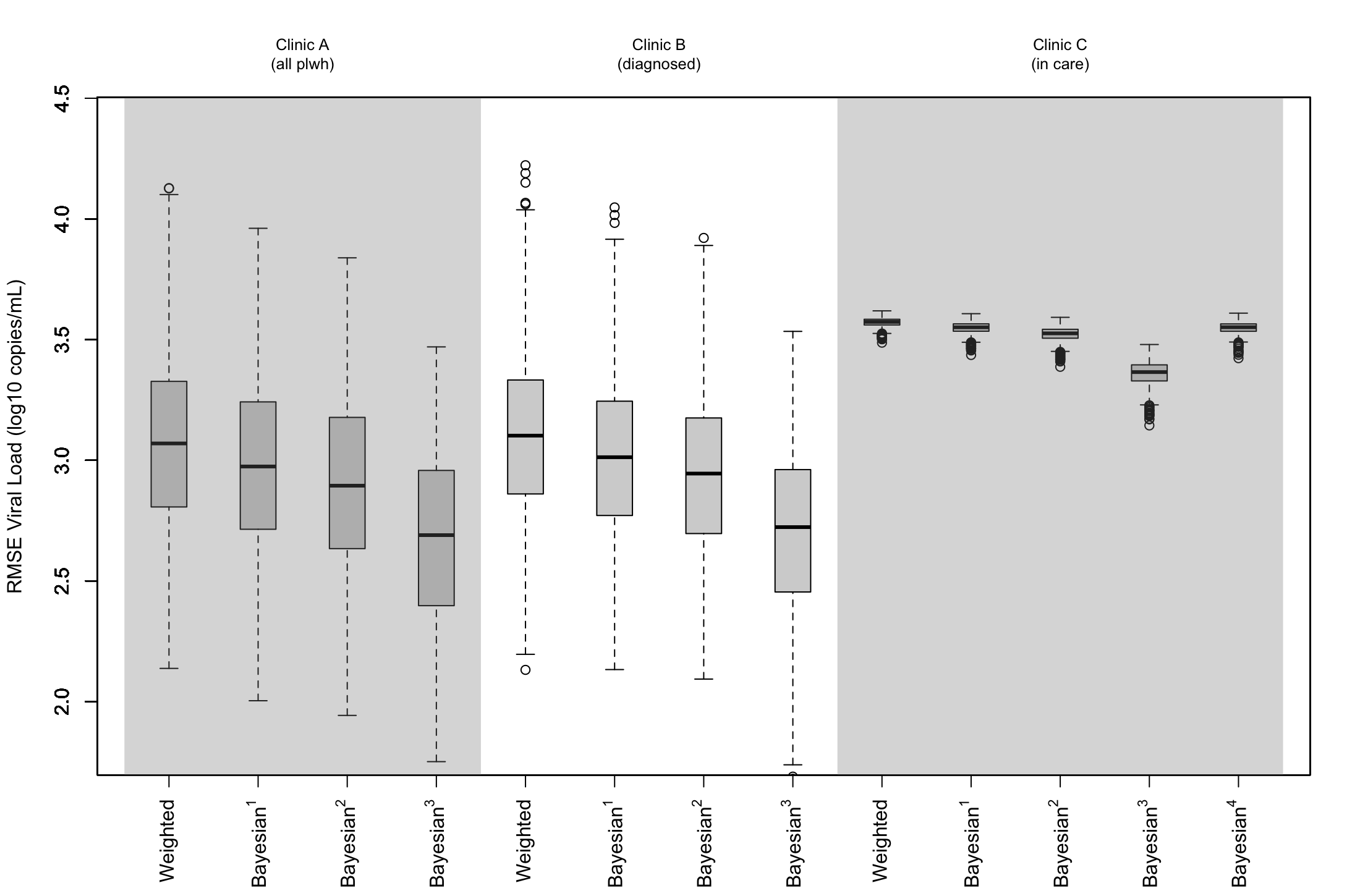

Supplement: Multimedia Appendix 2 [file ojphi_v16i1e58058_app2.png]

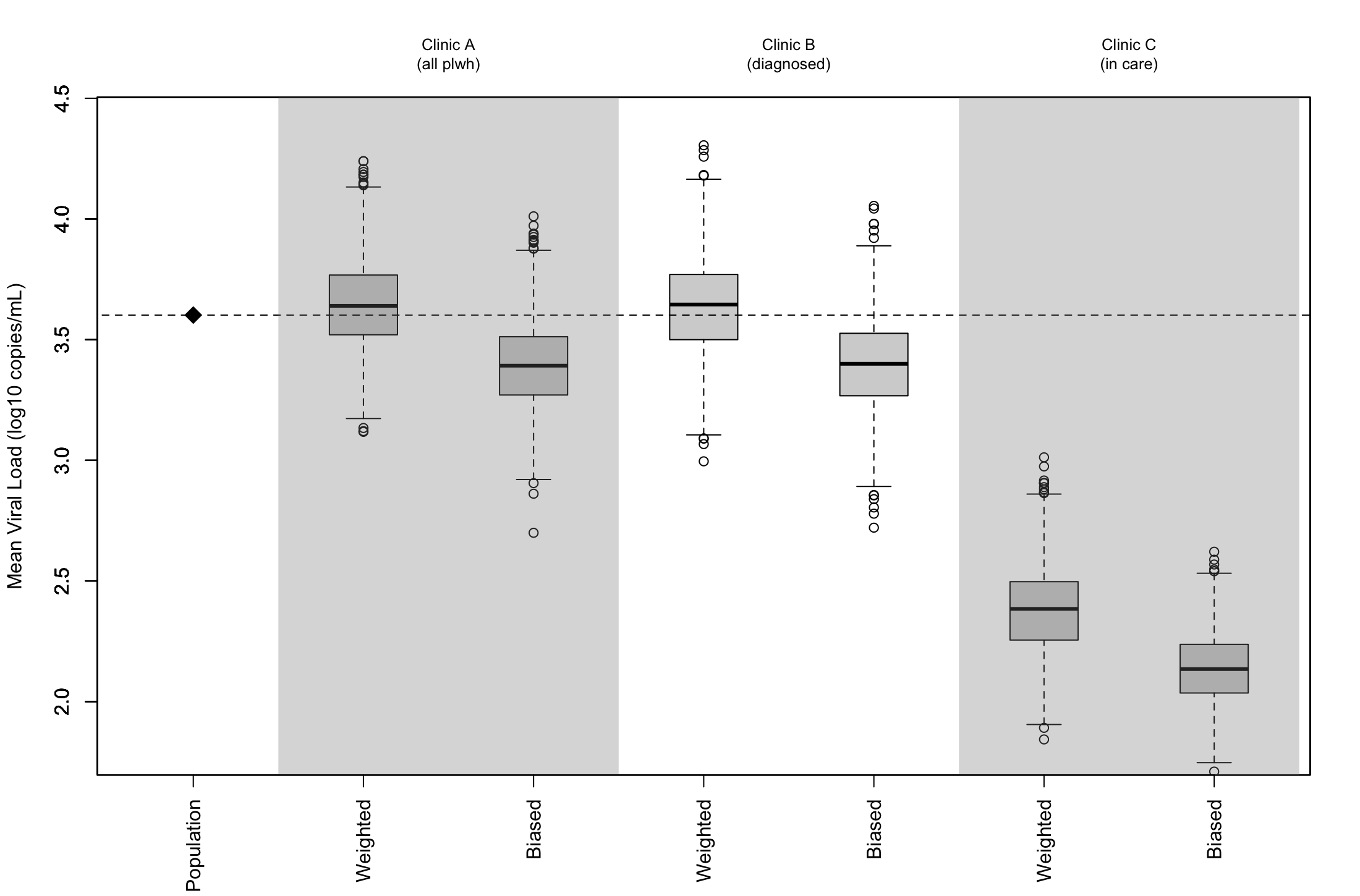

Supplement: Multimedia Appendix 3 [file ojphi_v16i1e58058_app3.png]

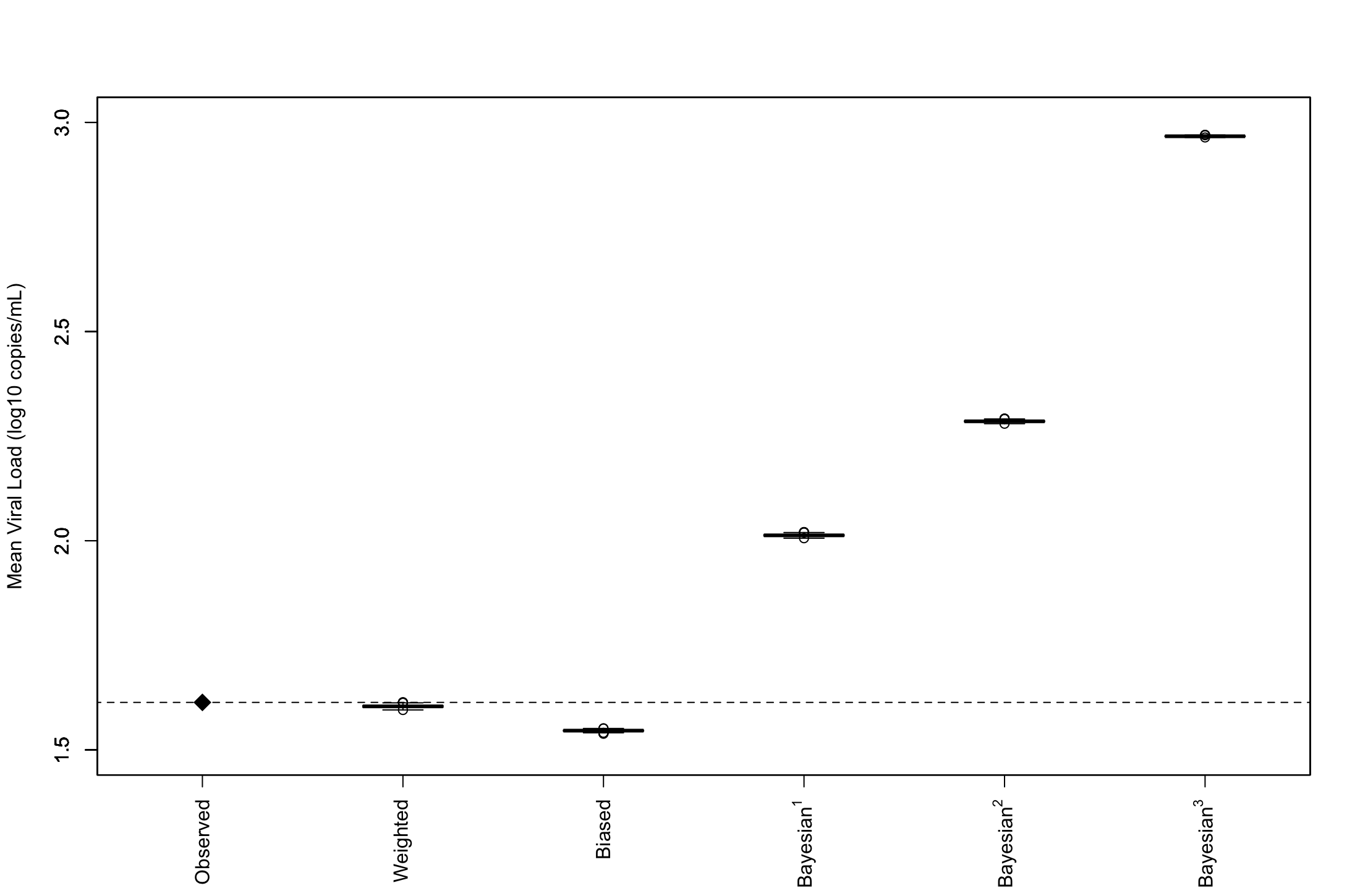

Supplement: Multimedia Appendix 4 [file ojphi_v16i1e58058_app4.png]

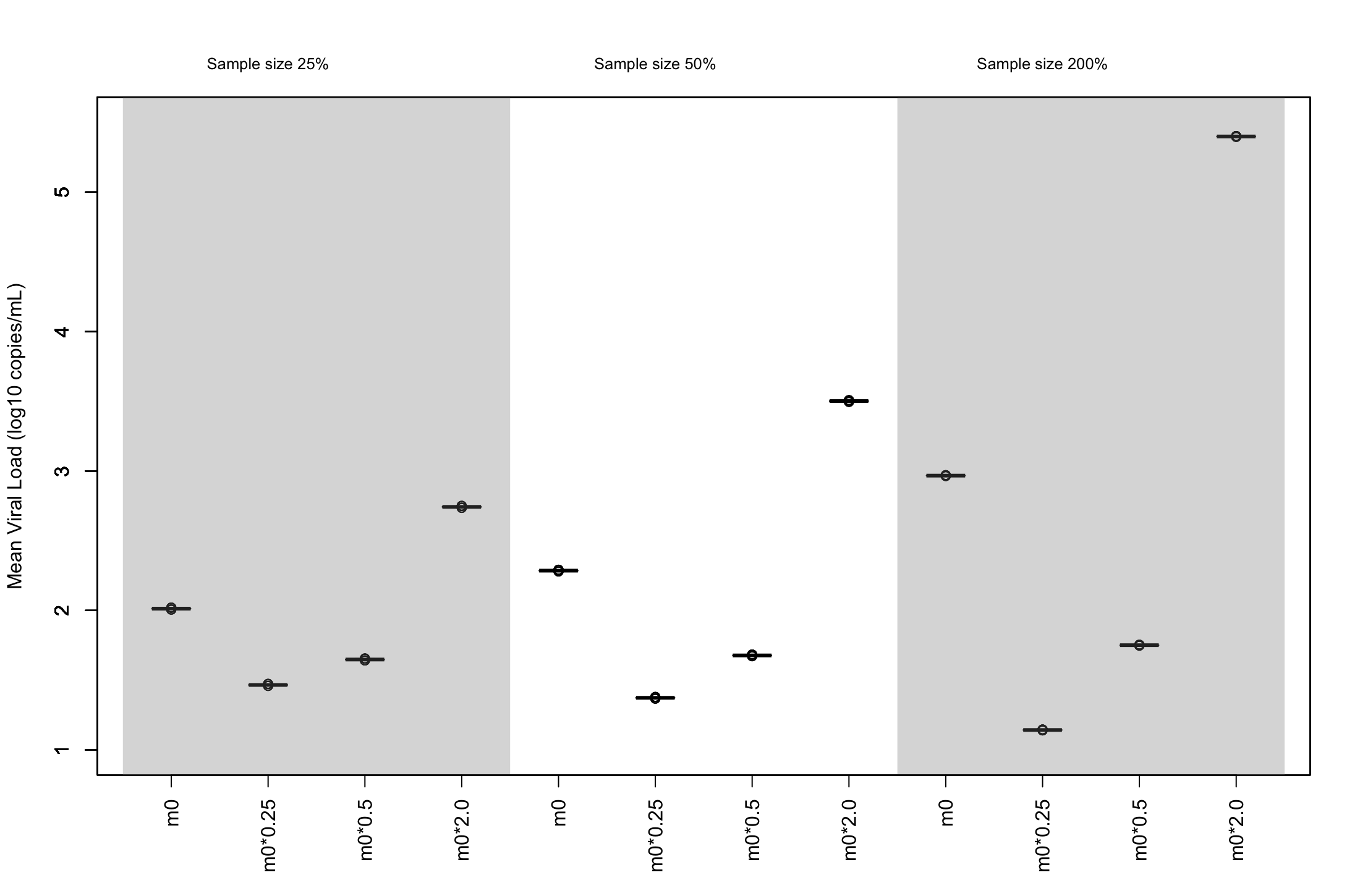

Supplement: Multimedia Appendix 5 [file ojphi_v16i1e58058_app5.png]
